# The Effect of Local Application of Tea Tree Oil Adjunctive to Daily Oral Maintenance and Nonsurgical Periodontal Treatment: A Systematic Review and Meta-Analysis of Randomised Controlled Studies

**DOI:** 10.3290/j.ohpd.b5458585

**Published:** 2024-06-12

**Authors:** Chenjiao Zhang, Bowen Liu, Jingchao Hu, Li Zhao, Han Zhao

**Affiliations:** a Dentist, Department of General, Beijing Stomatological Hospital, Capital Medical University, Beijing, China. Study concept and design, collected data and drafted the manuscript, gave final approval and agreed to be accountable for all aspects of the work.; b Oral Surgeon, Department of Oral and Maxillofacial Plastic Surgery, Beijing Stomatological Hospital, School of Stomatology, Capital Medical University, Beijing, China. Study concept and design, supported the manuscript working process, gave final approval and agreed to be accountable for all aspects of the work.; c Periodontologist, Department of Periodontics, Beijing Stomatological Hospital, School of Stomatology, Capital Medical University, Beijing, China. Critically revised the manuscript, gave final approval and agreed to be accountable for all aspects of the work.; d Prosthodontis, Department of Prosthodontics, Stomatological Hospital of Chongqing Medical University, Chongqing, China; Chongqing Key Laboratory of Oral Diseases and Biomedical Sciences, Chongqing, China; Chongqing Municipal Key Laboratory of Oral Biomedical Engineering of Higher Education, Chongqing, China. Performed the statistical analysis, gave final approval and agreed to be accountable for all aspects of the work.; e Periodontologist, Multi-disciplinary Treatment Center, Beijing Stomatological Hospital, School of Stomatology, Capital Medical University, Beijing, China. Contributed to study concept and design, collected data and drafted the manuscript, gave final approval and agreed to be accountable for all aspects of the work.; * Chenjiao Zhang and Bowen Liu contributed equally to this paper.

**Keywords:** dental plaque, meta-analysis, scaling and root planing, tea tree oil

## Abstract

**Purpose:**

To evaluate the efficacy of the adjunctive use of tea tree oil (TTO) for dental plaque control and nonsurgical periodontal treatment (NSPT).

**Materials and Methods:**

Three electronic databases were searched from 2003. The reference lists of the included articles and relevant reviews were also manually searched. Randomised controlled trials reporting the clinical outcomes of the topical use of TTO as an adjunct to daily oral hygiene or scaling and root planing (SRP) were included. Regarding the use of TTO as an adjunctive to daily oral hygiene, the primary outcome was plaque index (PI) reduction. Regarding the use of TTO as an adjunctive to SRP, probing pocket depth (PPD) reduction and clinical attachment level (CAL) gain were the primary outcomes. The secondary outcomes were adverse events.

**Results:**

Eleven studies were included for qualitative analysis, 9 studies were included for quantitative analysis, and 6 studies were included to examine the application of TTO mouthwash as an adjunctive to daily oral hygiene. In addition, three studies were included to analyse the subgingival use of TTO adjunctive to SRP at selected sites. The results indicated a nonsignificant improvement in PI reduction in the TTO mouthwash group compared with placebo. The incidence of adverse events was statistically significantly greater in the CHX group than in the TTO group. For subgingival use of TTO adjunctive to SRP, beneficial effects were observed in the TTO group compared with SRP alone in terms of PPD and CAL at both three and six months post-treatment. However, an unpleasant taste was reported in three out of four studies.

**Conclusion:**

There is a lack of strong evidence to support the beneficial effects of TTO. Studies with larger sample sizes and standardised evaluation criteria are needed to further demonstrate the clinical relevance of TTO.

Periodontal disease is considered a multifactorial inflammatory disease that originates from the accumulation of dental plaque at the gingival margin and develops as a consequence of the imbalance between the microbial load in periodontal tissue and the host immune response.^13,20,29,34,35^ Dental surfaces facilitate the development of highly complex polymicrobial biofilm communities, which form a dynamic ecosystem with host tissue. Disturbance of this dynamic ecosystem presents opportunities for oral microbial dysbiosis and the development of dental and periodontal diseases.^12,20,24,43,54^ The basis of periodontal treatment is the elimination or suppression of periodontal pathogenic microorganisms. The clearance of dental plaque can be achieved through mechanical periodontal treatment and can be maintained through careful daily oral hygiene.^42^ Local antimicrobial agents are usually administered as an adjunct to mechanical plaque control to disrupt the dental plaque biofilm structure and provide additional improvements in clinical outcomes.^17,20^ To date, chlorhexidine (CHX) is considered to be the most effective local antimicrobial agent and is widely used as an adjunct for periodontal treatment.^52,53^ Several reviews and meta-analyses have confirmed the effectiveness of the adjunctive use of CHX in periodontal therapy.^21,56^ CHX has long been regarded as the “gold standard” for local agent application associated with periodontal treatment.^23^

However, with the development of modern medicine, the evaluation of medical quality is not limited to clinical effectiveness; the comfort of patients during and after treatment has received increasing attention and has become an important indicator in the evaluation of medical quality. Discomfort during treatment and post-treatment adverse events have a considerable influence on patient compliance and adherence. Therefore, it is worth noting that the beneficial effects of CHX agents are accompanied by an array of adverse effects, such as taste changes, dental dyschromia, burning sensations and tongue discolouring.^15,16,28,36,39^ A meta-analysis by Costa et al^11^ in 2017 suggested that adjunctive use of CHX mouthwash combined with SRP resulted in only a slightly greater PD reduction than SRP alone and had a negligible effect on CAL. As clinicians, the balance between the minimal effect on clinical outcomes and the potential for tooth staining must be considered when using CHX as an adjunct to SRP to treat chronic periodontitis.^11^ In addition, several studies have indicated that allergies to CHX should also be considered.^54^ Therefore, a local adjunctive agent with an efficacy equal to that of CHX but with fewer side effects would be clinically ideal.

Due to increasing amount of attention devoted to the adverse effects of these agents, natural products have recently attracted an increasing amount of interest. A number of natural compounds have been used as adjuncts in the treatment of periodontal disease, such as *Matricaria chamomilla*, curcumin, green tea, triphala, and *Aloe vera*.^1,3,9,25,33^ These herbal agents have been used in nonsurgical periodontal treatment (NSPT) and subsequent daily oral maintenance. There is strong evidence supporting the adjunctive use of herbal products to achieve superior clinical outcomes compared with no adjunct, and outcomes comparable to that with CHX.^3,9,25^

Tea tree oil (TTO), which is derived from the paperbark tea tree, is widely used in medicinal products and daily care. The active ingredients of TTO are 1,8-cineole and terinen-4-ol, which exhibit antibacterial, antioxidant and anti-inflammatory activity.^5,26,27^ Groppo et al^14^ reported that TTO showed antimicrobial activity against mutans streptococci and other oral microorganisms. Mohammed et al^31^ indicated that *Melaleuca alternifolia* extract may be useful for preventing *P. gingivalis* infection. A systematic review in 2017 reported that TTO had effects comparable to those of 0.12% CHX in terms of reducing gingival inflammation and exhibiting antioxidant activity, which reduced host immune-inflammatory responses to pathogens.^7^ In contrast, a clinical trial performed by Soukoulis and Hirsch^48^ examined the use of TTO as a toothbrushing gel, showing that although TTO improved the gingival index and papillary bleeding index, it had no effect on plaque control. A review by Singh et al^45^ indicated that TTO was superior to CHX in terms of reducing signs of gingival inflammation, but CHX was superior to TTO in terms of inhibiting plaque formation. Because few clinical studies have focused on comparisons between TTO and CHX in the periodontal field, there is a lack of strong empirical evidence supporting the adjunctive use of TTO for periodontal-health maintenance and NSPT. In addition, although several studies reported that the adjunctive use of TTO was associated with fewer adverse events than the use of CHX,^36,39^ no systematic reviews have compared adverse events between the adjunctive use of TTO and CHX. The aim of this systematic review and meta-analysis was to comprehensively evaluate the benefits of the adjunctive use of TTO for dental plaque control and periodontal treatment based on clinical efficacy and the occurrence of adverse effects, as there is still no consensus on this topic.

## Materials and Methods

This systematic review was registered in PROSPERO under the ID CRD 42024525588.

### PICO Question

Is the local adjunctive application of TTO more effective than conventional interventions alone in healthy patients and those with periodontal disease?

- Population: Healthy people or patients with periodontal diseases.
- Intervention: Local adjunctive use of TTO for daily oral maintenance or NSPT.
- Comparison/Control: CHX, Placebo (PLB).
- Outcome: Clinical periodontal parameters, including plaque index (PI) change, probing pocket depth (PPD) reduction, clinical attachment loss (CAL) gain, and adverse events.

### Eligibility Criteria

The inclusion criteria for the studies were as follows: (1) randomised clinical trials (RCTs); (2) studies in which TTO was used as the main ingredient and compared with CHX or placebo; (3) studies reporting on multiple groups or different treatment modalities in which the results of TTO and CHX/placebo arms were reported; (4) studies in which at least one clinical periodontal parameter, including PI, PPD and CAL, was recorded; and (5) studies published in English. The exclusion criteria were as follows: 1) not RCTs; 2) duplicate publications; 3) unclear or complex ingredient combinations; (4) no CHX or placebo groups; and (5) no clinical results.

### Search Strategy

The review and meta-analysis were performed in accordance with the Preferred Reporting Items for Meta-Analysis (PRISMA) statement.^32^ Three independent reviewers (HZ, JCH and LZ) searched the PubMed, Embase and Cochrane Collaboration Library electronic databases for pertinent papers published between 2003 and 29 February 2024 to identify articles that examined the questions of interest. Furthermore, the reference lists of previous reviews and the included studies were manually searched.

### Study Selection

Two reviewers (HZ & JCH) independently reviewed the titles and abstracts.

In the first phase, studies that met all the inclusion criteria or did not meet any of the exclusion criteria were included for full-text screening.

In the second phase, full-text papers were assessed based on the eligibility criteria. In addition, the reasons for exclusion were recorded. Any disagreements were resolved by discussion among the three reviewers, and a consensus was reached through voting. The level of agreement between the reviewers was calculated using kappa statistics. Kappa values were classified as follows: ≤ 0 indicated no agreement, 0.01–0.20 indicated no to slight agreement, 0.21–0.40 indicated fair agreement, 0.41–0.60 indicated moderate agreement, 0.61–0.80 indicated substantial agreement, and 0.81–1.00 indicated perfect agreement.^30^ The search strategy for PubMed (adapted to the other databases) was as follows: (periodontitis OR gingivitis OR periodontal disease OR dental plaque OR dental biofilm OR dental deposits) AND (Herbal, OR Herbal Medicine, OR Herbalism, OR Phytotherapy, OR Herbal Therapy, OR Plant Extracts, OR Melaleuca alternifolia OR Tea Tree Oil, OR terpinenol-4).

### Primary and Secondary Outcomes

Two arms were identified according to the different applications of TTO: 1) adjunctive TTO for daily oral hygiene against dental plaque; in this arm, PI reduction was analysed as the primary outcome; and 2) subgingival application of TTO adjunctive to SRP in periodontitis; in this arm, PPD reduction and CAL gain were evaluated as the primary outcomes. The secondary outcome was adverse events.

### Risk of Bias Assessment

The quality of the included randomised clinical trials was independently assessed by two reviewers (HZ & CJZ) using the revised risk of bias assessment tool from the Cochrane Collaboration’s handbook, version 5.1.0.^18^ The included studies were evaluated based on the following seven criteria: random sequence generation, allocation concealment, blinding of participants and personnel, blinding of outcome assessment, incomplete outcome data, selective reporting, and other sources of bias. Each domain was judged as having a low risk of bias (seven “low risk” ratings), high risk of bias (one or more “high risk” ratings), or unclear risk of bias (one or more “unclear risk” ratings).

### Data Extraction

A standardised data extraction form was used by two independent reviewers (HZ & JCH). The following data were recorded: study design, participant characteristics, number of subjects, intervention and control, clinical outcomes, adverse events and length of follow-up.

### Data Synthesis

The meta-analyses were performed using RevMan version 5.3 (2014). The risk ratio and mean difference (MD) with 95% confidence intervals (95% CIs) were used for dichotomous and continuous data, respectively. The inverse variance method and a random-effects model were used due to the differences in variables between studies (different participant characteristics, periodontal diseases and interventions). Forest plots and funnel plots were generated, and the pooled effect was considered to be statistically significant at p < 0.05. Heterogeneity was evaluated using the I^2^ test and was categorised into four levels: 0%–25%, no heterogeneity; 25%–50%, low heterogeneity; 50%–75%, moderate heterogeneity; and 75%–100%, high heterogeneity.^19^

## Results

### Literature Selection

**Fig 1:**
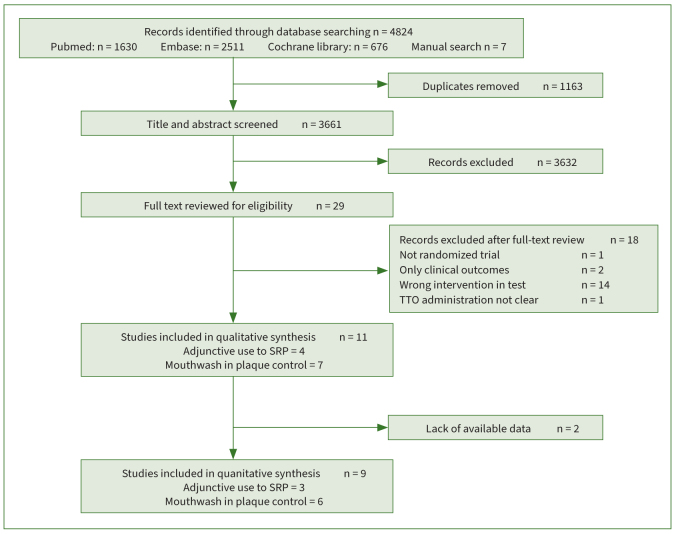
Study-selection flowchart based on PRISMA, with reasons for exclusion.

A total of 4824 studies were initially identified: 1630 from PubMed, 2511 from Embase, 676 from the Cochrane Library database, and 7 from a manual search. After the removal of duplicates (n =1163), 3661 articles were included for title and abstract screening. In this phase, 3632 papers were excluded. A total of 29 papers were included in the full-text screening. Eighteen studies were further excluded (Table 1), and 11 papers were ultimately included in the qualitative synthesis.^6,13,25,36-40,44,49,50^ The kappa value for inter-reviewer agreement was 0.84. Figure 1 shows the PRISMA study-selection flowchart as well as the reasons for exclusion.

**Table 1:** Reasons for exclusion of studies

N	Study title	Author	Reason for exclusion
1	Efficacy of an herbal antioxidant as an adjunct to nonsurgical periodontal therapy on procalcitonin levels in smokers with chronic periodontitis	Sravya et al (2019)	Wrong intervention, Oxitard
2	Effects of preprocedural mouth rinse on microbial load in aerosols produced during the ultrasonic scaling: A randomized controlled trial	Das et al (2022)	Herbal ingredients not clear, no clinical results
3	Evaluation of a hydrophobic gel adhering to the gingiva in comparison with a standard water-soluble 1% chlorhexidine gel after scaling and root planing in patients with moderate chronic periodontitis. A randomized clinical trial	Rusu et al (2017)	Wrong herbal ingredient
4	Efficacy of subgingival irrigation using herbal extracts on gingival inflammation	Pistorius et al (2003)	Herbal ingredients not clear
5	Efficacy of preprocedural mouth rinsing in reducing aerosol contamination produced by ultrasonic scaler: a pilot study	Gupta et al (2014)	Wrong herbal ingredient
6	A randomized, controlled clinical trial on the clinical, microbiological, and staining effects of a novel 0.05% chlorhexidine/herbal extract and a 0.1% chlorhexidine mouthrinse adjunct to periodontal surgery	Duss et al (2010)	Herbal ingredients not clear
7	Compare the effcacy of two commercially available mouthrinses in reducing viable bacterial count in dental aerosol produced during ultrasonic scaling when used as a preprocedural rinse	Shettey et al (2013)	No clinical results
8	Comparative evaluation of the effect of 0.2% chlorhexidine, 2% lemongrass oil, and 2% tea tree oil mouth rinse on salivary pH: An in vivo study	Manikandan et al (2021)	No clincial results
9	Antimicrobial effect of Melaleuca alternifolia dental gel in orthodontic patients	Santamaria Jr et al (2014)	Not randomised
10	Advantages of using toothpaste containing propolis and plant oils for gingivitis prevention and oral cavity hygiene in cleft lip/palate patients	Machorowska-Pieniążek et al (2021)	Complex herbal ingredient
11	Effect of oil pulling on plaque induced gingivitis: a randomized, controlled, triple-blind study	Asokan et al (2009)	Wrong herbal ingredient
12	A comparative study of antiplaque and antigingivitis effects of herbal mouthrinse containing tea tree oil, clove, and basil with commercially available essential oil mouthrinse	Kothiwale et al (2014)	Complex herbal ingredient
13	Comparative evaluation of antiplaque and antigingivitis effects of an herbal and chlorine dioxide mouthwashes: A clinicomicrobiological study	Siddeshappa et al (2018)	Unclear herbal ingredient (Hi-ora)
14	Comparison of a commercially available herbal and 0.2% chlorhexidine mouthrinse for prevention of oral malodor: A clinical trial	Mishra et al (2016)	Complex herbal ingredient (no TTO)
15	Effects of fixed orthodontic treatment and two new mouth rinses on gingival health: A prospective cohort followed by a single-blind placebo-controlled randomized clinical trial	Sobouti et al (2018)	Wrong herbal ingredient (Persica)
16	Clinical evaluation of chlorhexidine and essential oils’ adjunctive effects in subgingival ultrasonic instrumentation on periodontal parameters and halitosis	Tumer et al (2019)	Wrong herbal ingredient (Listerine)
17	The effects of a tea tree oil-containing gel on plaque and chronic gingivitis	Soukoulis et al (2004)	Not adjunctive
18	Comparison between tea tree oil and chlorhexidine mouth rinse in treatment of gingivitis induced by orthodontic treatment: a randomized control clinical study	Elmehy et al (2018)	The administration of TTO not clear

### Description of the Included Studies

Eleven randomised, placebo-controlled clinical trials met the inclusion criteria and were included in the qualitative analysis. Patient samples ranged from 15 to 160. Seven studies examined the use of TTO mouthwash as an adjunct to mechanical oral hygiene for dental plaque control.^5,25,36,38-40,49^ Three trials included patients with gingivitis,^38-40^ two studies included participants without periodontitis,^6,16^ one study did not report the type of periodontitis included,^46^ and one study included the oral health of schoolchildren only with a gingival score (GS) and a plaque score (PS) >1.^25^ All seven trials used TTO solution as the mouthwash. Five studies used both CHX solution and placebo as controls.^25,36,38,40,49^ Two studies used only a placebo as a control.^6,39^ The duration of TTO application ranged from 4 days to 4 weeks. Four studies reported clinical outcomes at 4–7 days,^6,25,36,49^ three studies reported outcomes at 2 weeks,^38-40^ and only one trial reported results at 4 weeks.^25^ All seven studies reported an improvement in the TTO group for at least one clinical parameter. Notably, for the plaque index (PI) evaluation, two trials showed no additional benefits after using TTO mouthwash,^36,40^ and one study showed greater improvement in the PI at 15 days but a minimal effect at 1 week.^38^ However, there is insufficient evidence that TTO provides additional benefits compared with CHX.

Four articles included an additional arm: the adjunctive subgingival use of TTO for NSPT.^13,37,44,50^ In all four studies, patients with chronic periodontitis were included. Among them, three studies used 5% TTO gel as an adjunct to PNST,^13,37,50^ where the gel was injected into periodontal pockets using a needle syringe immediately after SRP. One study prepared dental films of TTO that were inserted into the selected site on day 0 and day 7.^40^ In addition, two studies compared TTO + full-mouth scaling and root planing (FMSRP) and FMSRP alone;^13,50^ the other two studies used a split-mouth design and compared TTO + subgingival scaling and root planing (SRP) to SRP alone.^37,44^ One study used placebo agents as a control,^37^ and the other three studies used SRP alone as the control.^13,44,50^ The follow-up ranged from 1 month to 6 months. All four studies reported a statistically significant improvement in PPD reduction and CAL gain in the TTO +SRP group at different time points. A summary of the characteristics of the included studies is shown in Table 2.

**Table 2:** Summary of characteristics of included studies

	Study	Design	Number of participants			Type of periodontal disease	Grouping	Intervention				Clinical parameters	Clinical results	Adverse events		Follow-up
			Initial	Final	Age in years			Test	Control	Treatment strategy	Timing			TTO	CHX	
1	Elgendy et al (2013)	Intersubject parallel	40	40	20–60	CP, PPD5–8 mm, BOP (+)	TTO / SRP alone	5% TTO Gel	/	FMSRP	After SRP	PI, GI, PPD, CAL	TTO showed a statistically significant improvement of PPD, CAL and GI compared with SRP alone	Invasive and unpleasant taste	/	7 days, 1,3,6 months
2	Raut and Sethi (2016)	Split–mouth	15	15	30–60	CP, PPD > 5 mm or CAL > 4 mm	TTO / CoQ10 / PLB	5% TTO Gel; CoQ10 Gel	PLB Gel	SRP	After SRP	PI, PPD, CAL, SBI	TTO and CoQ10 showed statistically significant improvement of PPD, CAL, SBI and GI compared with PLB	Slight change in taste	/	7 days, 1 month
3	Siddabasappa et al (2020)	split–mouth	30	30	35–50	CP, PD 5–7 mm	TTO alone / TTO+SRP/SRP alone/ Control	TTO biofilm	/	SRP	day 0, 7	PI, GBI, CAL, PPD	The PI and GBI were statistically significantly reduced by all treatment modalities For PPD and CAL, the best result was obtained with SRP + TTO	/	/	7, 21 days, 3,6,9 months
4	Taalab et al (2021)	Intersubject parallel	30	30	25–50	CP, CAL 3–4 mm, BOP (+)	TTO+SRP/SRP alone	5% TTO Gel	/	FMSRP	After SRP	BOP, GI, PPD, CAL	Statistically significant improvement of clinical parameters from baseline, PPD showed no difference between groups, CAL and BI showed statistically significant difference between groups.	Unpleasant taste	/	1,3,6 months
5	Rahman et al (2014)	4*4 cross–over	20	20	22.55±1.79	No periodontitis	TTO/ CPC/ CHX/ PLB	1.5% TTO solution; 0.05% CPC solution	0.12% CHX solution; PLB solution	Mouth wash	5 days	PI, GI, tooth staining, other side effects	CHX effected statistically significant reduction of PI, but TTO did not; in all groups, IG decreased statistically significantly	2 (bitter taste and burning sensation)	9 (4 bitter taste, 2 burning sensation, 1 dry mouth, 2 tooth staining)	1 week
6	Scalvatori et al (2017)	4*4 cross–over	16	16	21–37	Gingivitis	TTO/CHX/essential oils/ PLB	1.5% TTO solution; essential oil solution	0.12% CHX solution; PLB solution	mouth wash	2 weeks	FMPS, FMBS, GI, tongue discolouration and patina	TTO showed statistically significant reduction in GI, FMBS, but not in FMPS	/	8 (burning sensation)	2 weeks
7	Casarin et al (2018)	2x2 cross–over	60	60	24.7±5.73	No periodontitis	TTO/CHX	0.3% TTO solution	0.12 CHX solution	mouth wash	4 days	QHPI, questionnaire of side effects	CHX showed lower level QHPI than TTO	Poor taste	Greater changes in taste	4 days
8	Ripari et al (2020)	RCT	42	42	>18	Gingivitis	TTO (22) / CHX (20)	100%TTO mouth wash diluted in 100ml water	0.12 CHX solution	mouth wash	2 weeks	PI, GI, GBI, PPD, dental dyschromia	Statistically significant reduction of all parameters in CHX and TTO groups	4 typical smells	4 dyschromia, 4 taste change,12 burning sensation	2 weeks
9	Kamath et al (2020)	Intersubject parallel	152	152	8–14	PS and GS >1	TTO/AV/CHX/PLB	0.20%	0.2% CHX solution/PLB solution	Mouth wash	4 weeks	PI, GI	Statistically significant reduction of PI and GI in CHX and TTO groups	/	/	4 weeks
10	Reddy et al (2020)	Intersubject parallel	90	90	12–16	Gingivitis	TTO/CHX/PLB	0.2%TTO	0.2% CHX solution/PLB solution	Mouth wash	15 days	PI, GI,	Statistically significant reduction of PI and GI in two test groups at 2 weeks, one week not	1 (altered taste and burning sensation)	0	1 week, 15 days
11	Srikumar et al (2022)	Intersubject parallel	160	158	19–59	Not reported	TTO /CHX/ PLB	“Desert Essence”	0.12 CHX solution/PLB	Mouth wash	1 week	PI, TCI	Statistically significant reduction of PI and TCI in TTO and CHX groups	/	/	1 week

Studies varied according to study design, different periodontal intervention, and different adjunctive drugs. The number of patients initially and finally was recorded. In addition, clinical outcomes, adverse events and follow–up period were recorded. RCT: randomised clinical trial; TTO: tee tree oil; CHX: chlorhexidine; CP: chronic periodontitis; PS: plaque score; GS: gingival score; SRP: subgingival scaling and planing; PI: plaque index; GI: gingival index; BOP: bleeding on probing; PPD: periodontal pocket depth; CAL: clinical attachment loss; GBI gingival bleeding index; PLB Placebo; FMPS full mouth plaque score; FMBS; full–mouth bleeding score; TCI: tongue coating index.

### Risk of Bias

All 11 studies described the randomisation methods. Five trials did not report on their allocation methods in detail.^13,36,37,39,44^ Additionally, five studies did not clearly describe the blinding method.^13,25,37,39,44^ Two studies did not report details about the participants who were lost to follow-up.^26,46^ Two trials did not present clear data on clinical outcomes.^40,44^ Overall, eight trials were considered to have an “unclear” risk of bias,^13,25,36,37,39,40,44,49^ and three trials were judged to have a “low” risk of bias. The risk of bias is presented in Table 3.

**Table 3:** Risk of bias assessment

Authors (year)	Random sequence generation	Allocation concealment	Blinding of participants and personnel	Blinding of outcome assessment	Incomplete outcome data	Selective reporting	Other bias	Risk of bias
Elgendy et al (2013)	○	?	?	?	○	○	○	Unclear
Raut and Sethi (2016)	○	?	?	?	○	○	○	Unclear
Siddabasappa et al (2020)	○	?	○	○	○	?	○	Unclear
Taalab et al (2021)	○	○	○	○	○	○	○	Low
Rahman et al (2014)	○	?	○	○	○	○	?	Unclear
Scalvatori et al (2017)	○	○	○	○	○	?	○	Unclear
Casarin et al (2018)	○	○	○	○	○	○	○	Low
Ripari et al (2020)	○	?	?	?	○	○	○	Unclear
Kamath et al (2020)	○	○	○	?	?	○	○	Unclear
Reddy et al (2020)	○	○	○	○	○	○	○	Low
Srikumar et al (2022)	○	○	○	?	?	○	v	Unclear

### Synthesis of Results

Eleven studies were included in the quantitative analysis, and two trials were excluded due to insufficient data.^25,44^ Six articles analysing adjunctive TTO for oral hygiene maintenance were included. The PI was evaluated as the primary outcome, and four studies were included in the quantitative analysis.^6,36,38,49^ Differences in PI reduction at one week between the TTO and PLB groups and between the TTO and CHX groups were analysed. For longer-term use of mouthwash (ie, 2.4 weeks), data were insufficient for meta-analysis. The incidence of adverse events between TTO and CHX was evaluated as a secondary outcome, and four studies were included.^36,38-40^ In addition, for subgingival application of TTO adjunctive to SRP in periodontitis, three articles were included in the quantitative synthesis,^13,37,50^ and PPD reduction and CAL gain at 1, 3 and 6 months were analysed as the primary outcomes. There was insufficient data on adverse events, so that no quantitative analysis was conducted.

### Adjunctive TTO in Oral Hygiene Maintenance

**Fig 2:**
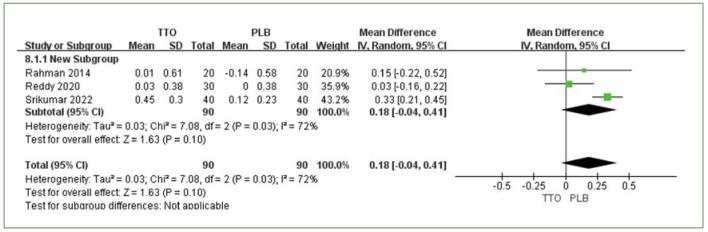
PI reduction comparing TTO and PLB at 1 week.

**Fig 3:**
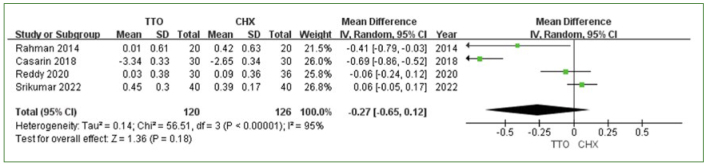
PI reduction comparing TTO and CHX at 1 week.

Three trials compared TTO and placebo (PLB),^36,38,49^ and four studies compared TTO vs CHX.^6,36,38,49^ PI reduction was the primary outcome in these studies. The meta-analysis revealed a slight, nonsignificant difference in the amount of PI reduction at one week between TTO and PLB (MD 0.18 [95% CI: -0.04–0.41]; p = 0.1), with moderate heterogeneity (I^2^ = 72%) (Fig 2). In the comparison between TTO and CHX, no statistically significant difference in PI reduction was observed (MD: -0.27 [95% CI: -0.65–0.12]; p = 0.18) (Fig 3). CHX mouthwash was slightly superior to TTO, and high heterogeneity was observed among the studies (I^2^ = 95%) (Fig 3).

### Adverse Events

**Fig 4:**
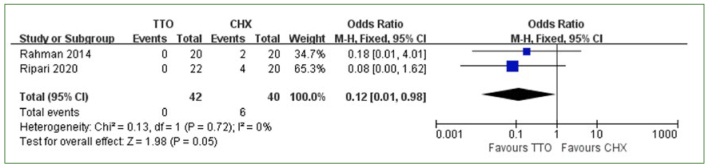
Dental dyschromia comparing TTO and CHX.

**Fig 5:**
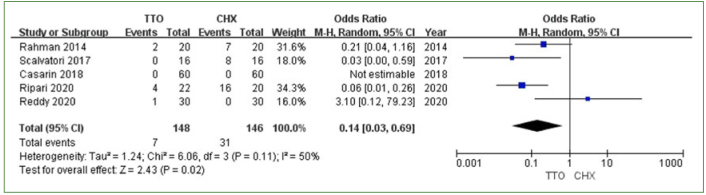
Feeling abnormal comparing TTO and CHX.

Five studies reported that adverse events occurred after the use of TTO and CHX.^6,36,39,40,49^ One study that used questionnaires was excluded from the meta-analysis of adverse events;^6^ in that study, the results of the questionnaire showed that CHX had a better taste than TTO but led to a greater change in taste.^6^ The other four studies were included in the meta-analysis of adverse events. There were 7 adverse events recorded in the TTO group and 35 adverse events recorded in the CHX group. Because of the complex classification of variable events, we included and summarised the data on dental dyschromia and feeling abnormal (taste alteration, nausea triggered by unpleasant taste, burning sensation). The results showed a statistically significantly lower risk of dental dyschromia (MD: 0.12 [95% CI: 0.01–0.98]; p = 0.05, I^2^ = 0%) (Fig 4) and feeling abnormal (MD: 0.14 [95% CI: 0.03–0.69]; p = 0.02, I^2^ = 50%) (Fig 5) in the TTO group.

### Adjunctive Use of TTO in SRP

**Fig 6:**
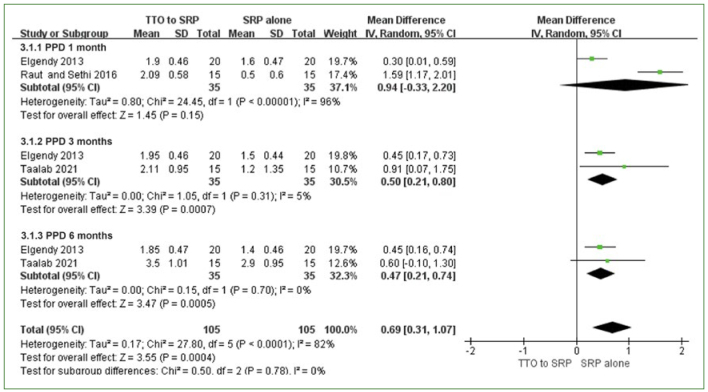
PPD reduction of TTO + SRP vs SRP alone at 1, 3, and 6 months.

**Fig 7:**
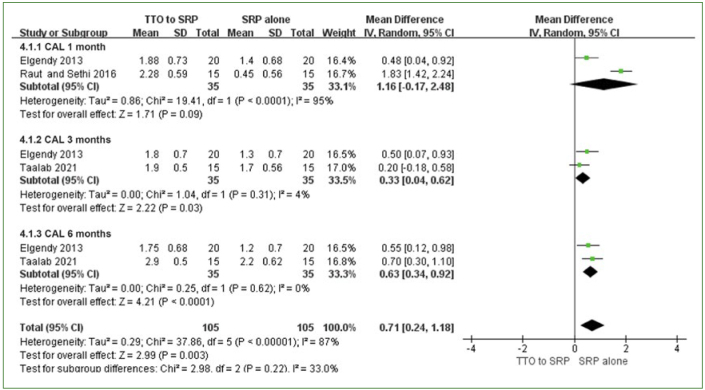
CAL gain of TTO + SRP vs SRP alone at 1, 3, and 6 months.

Three studies examined the adjunctive use of TTO in SRP.^37-39^ PPD reduction and CAL gain at 1, 3, and 6 months were analysed as the primary outcomes, and adverse events were analysed as the secondary outcomes. For PPD reduction and CAL gain, no statistically significant difference between the TTO and control groups was found at one month, with high heterogeneity (PPD: MD: 0.94 [95% CI: -0.33–2.20]; p = 0.15, I^2^ =96%; CAL: MD: 1.16 [95% CI: -0.17–2.48]; p = 0.09, I^2^ = 95%) (Figs 6 and 7). However, at 3 months and 6 months, a statistically significant effect was observed in the TTO group in terms of both PPD reduction and CAL gain (PPD at 3 months: MD: 0.50 [95% CI: 0.21–0.80]; p = 0.0007, I^2^ = 5%; PPD at 6 months: MD: 0.47 [95% CI: 0.21–0.74]; p = 0.0005, I^2^ = 0%; CAL at 3 months: MD: 0.33 [95% CI: 0.04–0.62]; p = 0.03, I2 = 4% CAL at 6 months: MD: 0.63 [95% CI: 0.34–0.92]; p<0.0001, I^2^ = 0%) (Figs 6 and 7). No heterogeneity was observed.

#### Adverse events

Three studies using 5% TTO gel reported slight adverse events associated with the adjunctive use of TTO in SRP.^13,37,50^ One trial reported a slight change in taste in the TTO group.^37^ The other two trials reported an unpleasant taste of the TTO gel.^13,50^ A study using TTO biofilms did not report the outcomes of adverse events.^44^ Due to the lack of data for adverse event assessment, a meta-analysis was not performed for this outcome.

## Discussion

This meta-analysis evaluated the effect of the topical adjuvant use of TTO on improving periodontal conditions. The classification for periodontal diseases was updated in a workshop co-sponsored by the American Academy of Periodontology (AAP) and the European Federation of Periodontology (EFP) in 2017.^8^ In addition to periodontal health and gingivitis, specific criteria for healthy periodontium and gingival inflammation in a reduced periodontium were defined after the completion of periodontitis treatment, which emphasises the need for more comprehensive maintenance.^8,51^ CHX is the “gold standard” local agent combined with periodontal treatment and maintenance, and due to the high incidence of adverse events, such as tongue and tooth staining as well as taste abnormalities, it cannot always be considered an ideal oral hygiene product for long-term use.^2^ Finding an alternative to CHX should be an important research direction. Research has indicated that both TTO and its nanoparticles act on virulence factors, negatively regulating the adhesion of microorganisms and decreasing biofilm formation.^10^ Hence, TTO is considered an adjunct to oral hygiene maintenance. This meta-analysis showed that the incidence of adverse effects was statistically significantly lower in the TTO group than in the CHX group, which compensated for the lack of CHX. Based on this evidence, adjunctive TTO may have the potential to replace CHX in daily oral hygiene. However, clinical studies using TTO gel as a toothpaste for toothbrushing have shown inconsistent results concerning PI score.^41,48^ It remains unclear whether TTO exerts clinical effects equal to those of CHX. In our review and meta-analysis, the use of TTO mouthwash for 1 week led to a nonsignificantly greater reduction in the PI than did the placebo. The moderate heterogeneity (I^2^=72%) reflected the inconsistency of the findings across the three included studies. Among them, one trial reported a statistically significant effect in the test group, and conversely, the other two studies suggested that the use of TTO mouthwash for one week did not reduce PI. However, in daily oral care, long-term results are more important, so that long-term, effective oral hygiene habits play a key role in healthy and stable periodontal conditions. This review included 3 articles evaluating the effect of TTO at 2 weeks, and the results are still contradictory. One study showed that the use of TTO for 2 weeks had no statistically significant effect on the PI, but the other two studies reported a statistically significant improvement in PI after using TTO mouthwash compared to the control. One of these studies reported that no statistically significant difference existed between TTO and PLB at one week. The last article examined the use of TTO mouthwash for four weeks, and the results revealed that this led to a statistically significant reduction in PI. Therefore, based on the available evidence, the effect of TTO mouthwash following daily oral hygiene against dental plaque formulation is not clear or conclusive. This conclusion was consistent with the findings of a previous systematic review published in 2017.^7^ Although the adjunctive use of TTO solution statistically significantly reduced the occurrence of adverse events, strong evidence on its clinical effects is lacking. Therefore, RCTs with longer follow-up durations across multiple time points are needed.

Four studies examined the subgingival use of TTO in SRP and were included in a qualitative review. Three of these studies were further included in a quantitative analysis. All four studies showed a statistically significantly improved clinical effect in the SRP+TTO group. However, among the four studies in which TTO gel was injected into periodontal pockets, three reported an unpleasant taste of TTO gel, which may influence patient acceptance of the treatment. Numerous local delivery agents are used in combination with SRP; CHX, minocycline and doxycycline are widely used as adjuncts to SRP, manifesting statistically significant clinical improvement compared to the control.^22,46^ Moreover, some studies which combined local agents with laser therapy following SRP reported a statistically significant improvement in clinical outcomes.^46,55^ No obvious adverse events were observed for these interventions. Overall, although the adjunctive use of TTO with SRP showed statistically significant effectiveness in terms of PPD and CAL, it did not present a statistically significant advantage over other agents or interventions.

## Limitations

To the best of our knowledge, this is the first meta-analysis focusing on the effects of the adjunctive use of TTO and classifying TTO application according to different clinical situations. Owing to the different study protocols, outcome assessments and evaluation parameters, it is difficult to guarantee the homogeneity of the included articles. In addition, most of the included studies were identified as having uncertain quality in the risk of bias assessment. The high heterogeneity in the one-week analysis of the PI and one-month analysis of the PPD and CAL reflected the inconsistency of the included articles. Therefore, a random-effects model was chosen to account for the variation that may have resulted from inconsistencies. However, the results of the random-effects model were relatively conservative, which may decrease statistical power between groups to a certain extent.

Moreover, although adjunctive TTO exhibits clinical benefits compared to SRP alone, comparisons between the adjunctive use of TTO and CHX are lacking.

## Conclusion

This meta-analysis revealed that the use of TTO adjunctive to daily oral hygiene statistically significantly reduced the incidence of adverse events compared to CHX, but did not statistically significantly reduce the PI. On the other hand, subgingival application of TTO in deep periodontal pockets following SRP led to statistically significantly reduced PPD and greater CAL gain, but the unpleasant taste of TTO gel should be borne in mind regarding patient compliance. The results of this study show a lack of strong evidence to support the benefit of adjunctive TTO. RCTs with larger sample sizes and consistent evaluation criteria are needed.
